# The Kiwifruit Allergen Act d 1 Activates NF-κB Signaling and Affects mRNA Expression of TJ Proteins and Innate Pro-Allergenic Cytokines

**DOI:** 10.3390/biom9120816

**Published:** 2019-12-02

**Authors:** Andrijana Nešić, Milena Čavić, Milica Popović, Milena Zlatanova, Raymond Pieters, Joost Smit, Marija Gavrović-Jankulović

**Affiliations:** 1 Department of Biochemistry, Faculty of Chemistry, University of Belgrade, 11000 Belgrade, Serbia; ananesic22@gmail.com (A.N.); la_bioquimica@chem.bg.ac.rs (M.P.); milena.zlatanova97@gmail.com (M.Z.); 2 Institute for Oncology and Radiology of Serbia, 11000 Belgrade, Serbia; milenaspasic@gmail.com; 3 Institute for Risk Assessment Sciences, Utrecht University, 3584 CM Utrecht, The Netherlands; R.H.H.Pieters@uu.nl (R.P.); J.J.Smit@uu.nl (J.S.)

**Keywords:** actinidin, epithelial cell-derived cytokines, food allergy, NF-ĸB, tight junction proteins

## Abstract

Impairment of the intestinal barrier is one of the key events in the initiation of the sensitization process in food allergy. The aim of this study was to explore the effects of kiwifruit allergen Act d 1 on intestinal permeability and tight junction protein (TJP) gene expression in vivo and to explore its potential to activate the NF-ĸB signaling pathway and to regulate expression of epithelial pro-allergenic cytokines. Influences of Act d 1 on TJP gene expression and pro-allergenic cytokines in the mouse intestine was analyzed by qPCR upon allergen administration by oral gavage. The effect on the in vivo intestinal permeability was assessed in ELISA by measuring the translocation of β-lactoglobulin (BLG) into circulation. The capacity of Act d 1 to activate the NF-ĸB pathway was tested in HEK293 cells by fluorescent microscopy and flow cytometry. Administration of Actinidin (Act d 1) increased intestinal permeability to the BLG. This was accompanied by changes in gene expression of TJP mRNA and pro-allergenic cytokines IL-25, IL-33, and thymic stromal lymphopoietin (TSLP) compared to the control. Act d 1 reduced TEER of the HEK293 monolayer, was positive in an NF-ĸB-reporter HEK293 cell assay, and induced secretion of TSLP. These findings shed more light on the molecular events in the sensitization process of kiwifruit but possibly also of other protease food allergens.

## 1. Introduction

The prevalence of food allergies has significantly increased and become a substantial health burden in developed countries [1]. A range of clinical manifestations affecting the gastrointestinal (GI) tract, skin, and lungs has been related to food allergy [2]. One of the main mechanisms underlying food allergy involves the breakdown of the GI barrier and immunological tolerance against ingested food allergens [1,3]. A pivotal role of the GI barrier is the maintenance of intestinal homeostasis and the first line of defense against various mechanical, chemical, and microbial stressors from the environment, including natural antigens occurring in food [4]. The capacity of epithelial cells to receive and decode signals from the environment and to employ received information in regulation of the responses of immune cells is critical for the maintenance of immune homeostasis [5]. 

In murine models of food allergy, oral exposure to antigen and an adjuvant stimulates gut epithelial cells to express the innate pro-allergenic IL-33, IL-25, and thymic stromal lymphopoietin (TSLP), which contribute to the development of acute reactions to food and promotion of Th2 response [1,6,7,8]. The integrity of the intestinal barrier and epithelial cells is maintained by intercellular junctional complexes composed of tight junctions (TJs), adherens junctions, and desmosomes. TJs are complex structures composed of more than 40 proteins, which represent signaling platforms that establish cell polarity, transmit signals into nuclei, and modulate gene expression [9]. Among a range of proteins that are connected to a junctional plaque, Rho GTPases are central components of signaling pathways that guide junction assembly and polarization as well as are involved in the mechanisms by which junctions transmit a signal to the cell interior [10]. Distinct Rho proteins have been involved in the regulation of the NF-ĸB transcription factor, which is involved in preserving intestinal immune homeostasis and has a role in the development of inflammatory intestinal diseases [11,12]. Activation of the NF-ĸB signaling pathway and release of epithelium-derived IL-33, TSLP, and IL-25 are observed in airway epithelium during the initiation of type 2 immune response in respiratory mucosa [13] and in food allergy [14]. The above would suggest that there is a direct link between TJ functionality, NF-ĸB, and sensitization. However, there is no evidence linking these sensitization events to kiwifruit allergens and NF-ĸB signaling pathway, so far.

Since 1981, there has been an increasing number of reports on kiwifruit allergy. It has become evident that kiwifruit allergy is frequently associated with grass and birch pollen allergies [15,16]. Children, however, are often mono-sensitized to kiwifruit, suggesting a role of primary digestive tract sensitization [17]. Thirteen allergens have been identified in kiwifruit (www.allergen.org), but cysteine protease Actinidin (Act d 1) is considered the major allergen, representing 50% of the total soluble protein content [16]. 

The aim of this study was (i) to evaluate the effects of Act d 1 on the TJ proteins in terms of their mRNA expression in mice intestine, (ii) to analyze mRNA expression levels of IL-25, IL-33, and TSLP in vivo and in vitro, and (iii) to test the potential of Act d 1 in activating NF-ĸB signaling pathway.

## 2. Materials and Methods 

### 2.1. Allergen Preparation

Actinidin (Act d 1) was purified from fresh kiwifruit by two consecutive ion-exchange chromatographies as previously described [18,19]. The purity of the protein preparation was estimated to be >97% as observed by SDS–PAGE. The proteolytic activity of the purified enzyme was determined by using an enzymatic assay with casein [20]. Before the treatment, Act d 1 was either activated in cell culture medium for 1 h at 37 °C for in vitro experiments or with 0.2 mM cysteine for in vivo experiments. For inactivation, cysteine protease inhibitor E-64 (Sigma-Aldrich, St. Louis, MO, USA) was added to the Act d 1 solution in an equimolar ratio (v/v).

### 2.2. Animals 

C57BL/6 mice (*n* = 6/group) (Charles River Laboratories, Maastricht, The Netherlands) were housed under controlled conditions in standard laboratory cages. All in vivo experiments were approved by the Ethics Committee for Animal Experiments (AVD108002016394) and were performed in compliance with governmental and international guidelines on animal experimentation. 

### 2.3. The Dosage Regimen of Act d 1 and Permeability Assay

The effect of Act d 1 on the in vivo permeability across the intestinal mucosa was assessed by measuring the translocation of β-lactoglobulin (BLG) into mouse circulation upon oral gavage. A control group of mice (*n* = 6) received 350 μL of PBS with 0.2 mM L-cysteine. The second group (*n* = 6) received 350 μL of E-64 inactivated Act d 1 (5 mg/mL in PBS), and the third group (*n* = 6) received active Act d 1 350 μL (5 mg/mL in PBS) by oral gavage. After 24 h, mice from all three groups received BLG by oral gavage (500 mg/kg BW). Blood samples (~150 μL) were drawn from the cheek vein 30 min after BLG administration. Following centrifugation (3000× *g*, 10 min), sera were collected and diluted 1:1 (v/v) in PBS. ELISA capture was performed for quantification of BLG in mice sera according to the manufacturer’s instructions (Bethyl Laboratories, Inc., Montgomery, TX, USA).

### 2.4. Isolation of RNA and qRT-PCR of Mouse Intestinal Samples

For mRNA studies, the mouse intestine was flushed with cold PBS and separated into the following segments: proximal small intestine (first centimeter of the proximal part of the jejunum, 2 cm after the stomach) and distal small intestine (final centimeter before the ileum–caecum–colon junction) and colon. These intestinal samples (1 cm) were snap-frozen in liquid nitrogen and stored at −80 °C. Total RNA was extracted from each tissue sample (50 mg) using TRI Reagent^®^ and homogenized using a tissue lyser (Qiagen, Hilden, Germany) for 1 min at 25 Hz. The complementary DNA (cDNA) was prepared from total RNA (2 µg) by using random primers and MultiScribe™ Reverse Transcriptase from the High-Capacity cDNA Reverse Transcription kit (Applied Biosystems, Foster City, CA, USA). Gene expression analysis was assessed by quantitative real-time PCR (*q*RT-PCR). PCR reactions were performed using the ABI Prism 7500 Sequence Detection System (Applied Biosystems, Foster City, CA, USA). The reaction mixture for the *q*RT-PCR, containing 1 µL undiluted cDNA and 5 µL Maxima SYBR Green/ROX qPCR Master Mix (2×) (Thermo Fisher Scientific, Waltham, MA, USA), forward and reverse primers (final concentration of 300 nM for each primer), and sterile deionized water, was prepared according to the manufacturer’s instructions. PCR cycle parameters were as follows: general denaturation at 95 °C for 3 min, 1 cycle, followed by 40 cycles of 95 °C for 20 s, annealing temperature (AT) for 30 s, and elongation at 72 °C for 30 s. Gene-specific primers for CLDN1, CLDN2, CLDN3, CLDN4, OCLN, ZO protein 1 (ZO1), E-cadherin, IL-25, IL-33, and TSLP, (Table 1) were derived from the U.S. National Center for Biotechnology Information (NCBI, Bethesda, MD, USA) GenBank and were purchased from Invitrogen (Paisley, UK). The mRNA quantity was calculated relative to the expression of the reference gene (β-actin) and analyzed by the delta-delta-Ct method.

### 2.5. HEK293 Cell Culture, RNA Isolation, and Gene Expression Analysis

Human embryonic kidney epithelial HEK293 adherent cells (passages 15–25) were cultivated in Minimum Essential Medium (MEM, Sigma-Aldrich, St. Louis, MO, USA) supplemented with 10% heat-inactivated fetal bovine serum (FBS), penicillin (100 IU) and streptomycin (100 µg/mL) (Sigma-Aldrich, St. Louis, MO, USA) and in 200 mM L-glutamine (Sigma-Aldrich, St. Louis, MO, USA) in an atmosphere of 5% CO_2_ at 37 °C. Cells passaging were performed by using 1 × trypsin/EDTA solution (Sigma-Aldrich, St. Louis, MO, USA). Each experiment contained untreated monolayers as control to allow normalization of the data and statistical analysis. 

For transepithelial electrical resistance (TEER) measurement, HEK293 cells (0.6 × 10^5^ cells per well) were grown on polycarbonate cell culture inserts (0.4 µm diameter pore) in 24-well plates (Nunc, Roskilde, Denmark) at 37 °C in 5% CO_2_. The cells were allowed to grow for three days to reach 80–90% confluence. The cell monolayers were treated either with activated Act d 1 (1 mg/mL) or with E-64-inactivated Act d 1 (1 mg/mL) as previously described [21]. Act d 1 samples were applied onto the apical side of the cell monolayer, and TEER was measured by using a Millicell ERS meter (Millipore, Bedford, MA, USA). The results represent mean ± standard error (SEM) of three independent experiments. Untreated HEK293 cell monolayers were used as control.

HEK293 cells were seeded at density 0.2 × 10^6^ cells/well (12 well plate) and cultivated. Before the Act d 1 treatment, cells were washed with PBS and incubated in serum-free medium for 1 h. Cells were treated with activated or inactivated Act d 1 (1 mg/mL) for 6 h. Thereafter, the medium was removed and cells were washed with cold PBS and collected by centrifugation (800 rpm, 7 min). Isolation of RNA and evaluation of gene expression were performed as previously described [21]. GAPDH was used as a housekeeping gene to allow normalization of mRNA levels between samples. Gene-specific primers for human genes are listed in Table 2.

### 2.6. ELISA Detection of TSLP

ELISA capture assay was employed for the detection of pro-inflammatory cytokine TSLP in accordance with the manufacturer’s instructions (e-Bioscience, San Diego, CA, USA). Supernatants from HEK293 cells were collected after 6 h of treatment with Act d 1 (1 mg/mL). ELISA Maxisorp immunoplates (Nunc™, Reskilde, Denmark) were employed for cytokine detection as previously described [22].

### 2.7. NF-ĸB-GFP Reporter Assay

For transient transfection, HEK293 adherent cells with epithelial morphology were grown in T-75 flask (Nunc™ EasYFlask™ Cell Culture Flasks, Thermo Fisher Scientific, Waltham, MA, USA) and cultivated in Minimum Essential Medium (MEM, Sigma-Aldrich, St. Louis, MO, USA) supplemented with 10% heat-inactivated fetal bovine serum (FBS), penicillin (100 IU), and streptomycin (100 µg/mL) (Sigma-Aldrich, St. Louis, MO, USA) and in 200 mM L-glutamine (Sigma-Aldrich, St. Louis, MO, USA) in 5% CO_2_ at 37 °C. Before transfection, cells were passaged using 1 × trypsin/EDTA solution (Sigma-Aldrich, St. Louis, MO, USA) and seeded in complete growth medium containing serum and antibiotics in 96 well-plate (4 × 10^4^ cells/well) or glass coverslips in 24-well plate (8 × 10^4^ cells/well).

Cells were cultured until they reach ~80% confluence. On the day of transfection, 0.2 µg/well (or 0.4 µg/coverslip) of an appropriate DNA (Cignal NF-ĸB-GFP Pathway Reporter Assay Kit, Qiagen, Hilden, Germany) was diluted with medium without serum, antibiotics, and glutamine (Opti-MEM I Reduced Serum Media, Thermo Fisher Scientific, Waltham, MA, USA) to a total volume of 50 µL/well (60 µL/coverslip). Then, 0.75 µL/well (1.5 µL/coverslip) of Attractene Transfection Reagent (Qiagen, Hilden, Germany) was added to the DNA solution mix and incubated for 20 min at RT. The medium was replaced, and the transfection complex was added dropwise onto the cells. After 48 h of incubation, cells were treated with activated and E-64 inactivated Act d 1 (1 mg/mL). Treatment was performed for 24 h at normal growth conditions. Activation of the NF-ĸB-GFP was assessed by flow cytometry and by fluorescence microscopy.

For flow cytometry analysis, cells were detached with 1 × trypsin/EDTA solution (Sigma-Aldrich, St. Louis, MO, USA), centrifuged (2000 rpm, 5 min), and resuspended in 1 × PBS. GFP fluorescence intensity (10^4^ events per sample) was measured using FACS Calibur (BD Biosciences, San Jose, CA, USA). A blue solid-state 200-mW laser at 488 nm was used for excitation. The emission was detected with a 525-nm filter (FL1). The positive cells were gated on the SSC, FL1H plot. Values were expressed as the percentage of GFP fluorescence with respect to the negative control.

Act d 1 activation of NF-ĸB-GFP in transfected HEK293 cells was visualized by fluorescence microscopy. Cells on coverslips were fixed with 4% paraformaldehyde (Fisher Scientific, Waltham, MA, USA) in 1 × PBS for 15 min at RT. After washing with PBS (3×), cells were mounted on glass slides and examined by a fluorescence microscope (Opto-Edu, Beijing, China). Images were acquired by an objective with 60× magnification. For visualization, ImageView software was used and images were further processed using Adobe Photoshop CS5 (Adobe Systems Incorporated, San Jose, CA, USA).

### 2.8. Statistical Analysis

All in vivo data analyzed by the Shapiro–Wilk test (*n* < 50) revealed normal distribution within the groups. For in vivo permeability assay, independent t-test was employed. For gene expression analysis two-way analysis of variance (ANOVA) with a Bonferroni post hoc test to adjust the *p*-value for multiple comparisons was used. One-way ANOVA was used for ELISA and transfection assay. The results were considered statistically significant if *p* < 0.05. The analysis was performed using GraphPad Prism 5.0 (GraphPad, La Jolla, CA, USA).

## 3. Results

### 3.1. In Vivo Exposure to Act d 1 Leads to Increased Intestinal Permeability in C57BL/6 Mice

**Figure 1:**
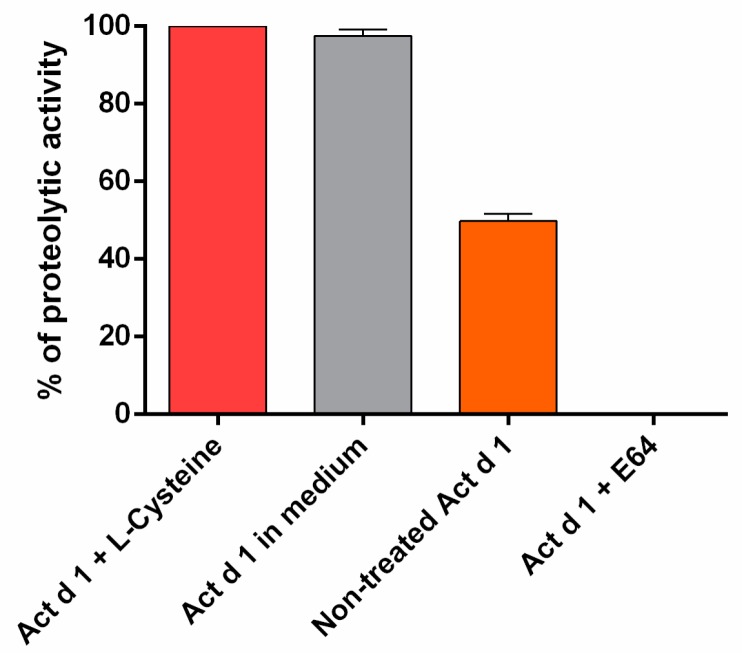
Proteolytic activity of Actinidin (Act d 1) preparation was assessed in a protease enzymatic assay with casein as a substrate: Results are expressed as mean ± SEM of three independent experiments, each performed in triplicate.

Proteolytic activity of Act d 1 preparation was tested in a protease enzymatic assay with casein as a substrate (Figure 1). Four samples of Act d 1 were analyzed: (a) Act d 1, (b) Act d 1 activated in the phosphate buffer pH 6.6 with L-cysteine, (c) Act d 1 activated in the cell culture medium, and (d) Act d 1 inactivated with E-64 inhibitor. Act d 1 revealed 55% of caseinolytic activity when compared to Act d 1 activated with L-cysteine (100%) and with Act d 1 activated with cell culture medium (98%). The proteolytic activity of the E-64-inhibited sample of Act d 1 was undetectable in the assay.

**Figure 2:**
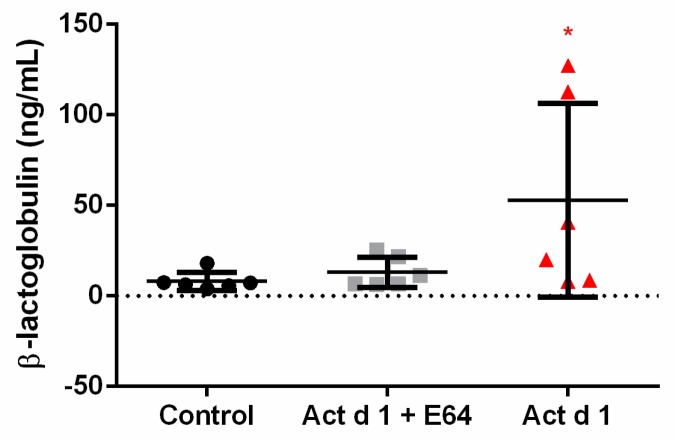
Act d 1, but not inactivated Act d 1, increased intestinal permeability in C57BL/6 mice. At 24 h after oral administration of Act d 1 or inactivated Act d 1 (0.35 mL, 5 mg/mL), mice were orally administered with β-lactoglobulin (BLG; 500 mg/kg BW). Thirty minutes after BLG gavage, sera were collected to analyze the presence of BLG. Sample means of the three mice groups were compared using an independent *t*-test; *n* = 6 animals/group. * *p* < 0.05, compared to control.

To test permeability in vivo, mice were orally challenged with β-lactoglobulin after Act d 1 treatment (Figure 2). Act d 1 induced an increase in intestinal permeability since increased levels of BLG were detected in sera of mice treated with activated Act d 1 but not of mice treated with E64-inactivated Act d 1.

### 3.2. Act d 1 Differentially Upregulates mRNA Expression of TJ Proteins in Distinct Segments of Mouse Intestine

**Figure 3:**
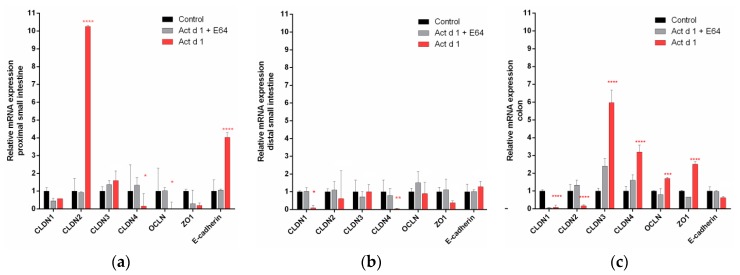
Effect of Act d 1 on mRNA expression of tight junction (TJ) proteins in different parts of the mouse intestine: proximal small intestine (**a**), distal small intestine (**b**), and colon (**c**). Mice were orally exposed to PBS (control), E64-inactivated Act d 1, and activated Act d 1. At 24 h, intestinal tissue was dissected, homogenized and mRNA was isolated for qRT-PCR analysis. Results are expressed as mean ± SEM relative mRNA expression and analyzed using a two-way analysis of variance (ANOVA); *n* = 5 animals per group. * *p* < 0.05; ** *p* ≤ 0.01, *** *p* ≤ 0.001, **** *p* ≤ 0.0001, compared to control.

The effect of Act d 1 on TJ gene expression in vivo was analyzed by mRNA levels of CLDN1, CLDN2, CLDN3, CLDN4, OCLN, ZO1, and E-cadherin in the proximal and distal small intestine and in the colon. The expressions of CLDN2 and E-cadherin were remarkably increased in the proximal part of the small intestine, while the expressions of CLDN4 and OCLN decreased (Figure 3a). In the distal part of the intestine, CLDN1 and CLDN4 were decreased (Figure 3b). In the colon, CLDN1 and CLDN2 expression were downregulated whereas the expressions of CLDN3, CLDN4, OCLN, and ZO1 increased (Figure 3c). Clearly, Act d 1 remarkably changed the expression levels of TJ proteins in the proximal small intestine and in the colon. The mRNA expression of TJ proteins and E-cadherin remained almost unaffected in the distal small intestine.

### 3.3. Act d 1 Induces Upregulation of mRNA for the Innate Pro-Allergenic Cytokines in Vivo

**Figure 4:**
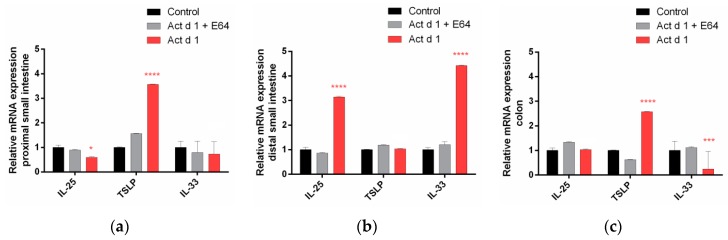
Act d 1 induced upregulation of mRNA for the innate pro-allergenic cytokines in different parts of the mouse intestine: (**a**) proximal small intestine, (**b**) distal small intestine, and (**c**) colon. Mice were orally exposed to PBS (control), E-64 inactivated Act d 1, and activated Act d 1. After 24 h, intestinal tissue was dissected and homogenized and mRNA was isolated for qRT-PCR analysis. Results are expressed as mean ± SEM relative mRNA expression; *n* = 5 animals per group. Results are expressed as mean ± SEM and analyzed using a two-way analysis of variance (ANOVA). * *p* < 0.05; *** *p* ≤ 0.001, **** *p* ≤ 0.0001, compared to control.

To explore the ability of Act d 1 to elicit an early inflammatory response, gene expression of the pro-allergenic epithelial cell-derived cytokines IL-25, IL-33, and TSLP were measured in distinct segments of mouse intestine (Figure 4). The level of TSLP mRNA was significantly increased in the proximal small intestine (Figure 4a), whereas IL-25 and IL-33 mRNA were upregulated in the distal part of mice intestine (Figure 4b). In the colon, the expression of TSLP was increased whereas IL-33 was downregulated (Figure 4c).

### 3.4. Act d 1 Affects HEK293 Monolayer Transepithelial Electrical Resistance

**Figure 5:**
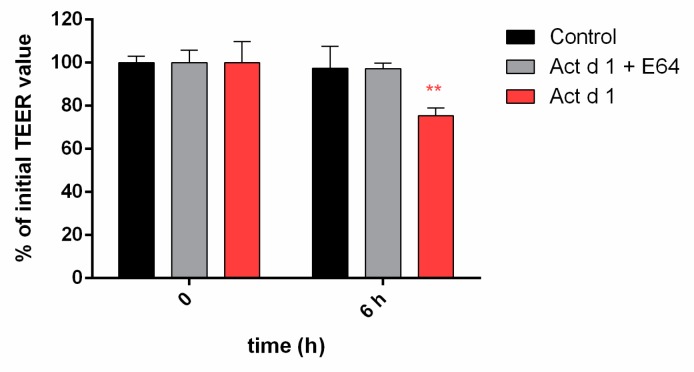
Active Act d 1 induced a decrease in transepithelial electrical resistance (TEER) values of HEK293 monolayers. HEK293 cells were grown on inserts, and the effect of activated Act d 1 and E64-inactivated Act d 1 on monolayer integrity was tested after 6 h. Results are expressed as mean ± SEM percentage of the initial value of three independent experiments performed in triplicate and analyzed using a two-way analysis of variance (ANOVA); ** *p* ≤ 0.01 compared to the initial TEER value (100%).

For enzyme activation, lyophilized Act d 1 was dissolved in cell culture medium and incubated for 1 h while inactive enzyme was obtained by Act d 1 incubation with E-64 inhibitor. Effects of Act d 1 proteolytic activity on the HEK293 cell monolayers integrity was assessed by measuring transepithelial electrical resistance (TEER) at 0 and 6 h of the treatment. Control cells maintain the basal TEER (98%) over the 6 h period, while Act d 1 treatment reduced TEER to 75% of control (** *p* ≤ 0.01). The TEER of monolayers exposed to E-64-inhibited Act d 1 was not significantly changed up to 6 h of treatment, indicating that the observed effect was protease dependent (Figure 5).

### 3.5. Act d 1 Activates the NF-ĸB Signaling Pathway and Upregulates the Innate Pro-Allergenic Cytokines In Vitro

**Figure 6:**
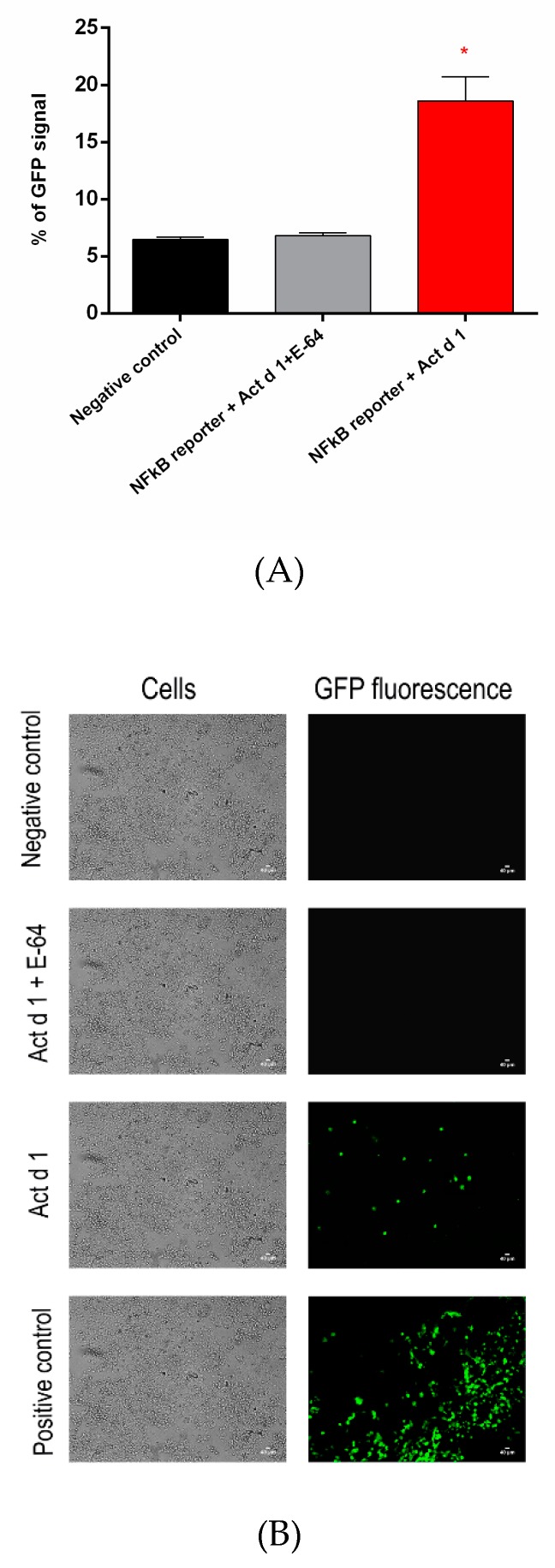
(**A**), Quantification of GFP fluorescence as a consequence of NF-ĸB signaling in HEK293 cells upon Act d 1 treatment: HEK293 cells were transiently transfected with NF-ĸB-GFP plasmid for 48 h and, thereafter, were stimulated with activated and inactivated Act d 1. GFP expression was evaluated by flow cytometry after 24 h. The cell population was gated using SSC versus FL1-H dot plot and then represented as a histogram plot. Values are presented as mean ± SEM and analyzed using a one-way analysis of variance (ANOVA). * *p* < 0.05, compared to control. (**B**) Visualization of Act d 1-induced activation of NF-ĸB signaling by detecting GFP expression with fluorescence microscopy: HEK293 cells were grown on glass coverslips, transfected with NF-ĸB-GFP plasmid, and treated with Act d 1 (activated or E-64 inactivated). After fixation, cells were mounted on glass slides and examined by fluorescence microscopy.

Act d 1 activation of NF-ĸB signaling in transfected HEK293 cells was detected as induced GFP expression by flow cytometry (Figure 6A) and fluorescence microscopy (Figure 6B) in comparison to negative control and E-64-inactivated Act d 1.

**Figure 7:**
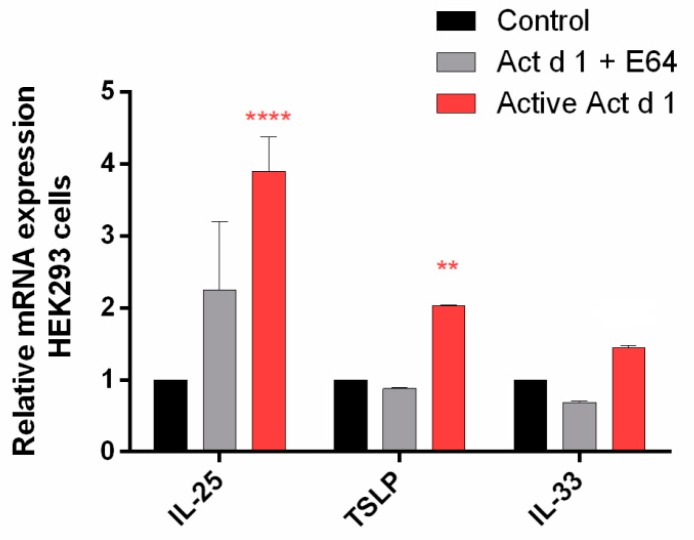
Act d 1 induced the upregulation of mRNA for the innate pro-allergenic cytokines in vitro. HEK293 cells were treated with activated Act d 1 or E-64-inactivated Act d 1. Results are expressed as mean ± SEM and analyzed using a two-way analysis of variance (ANOVA). ** *p* ≤ 0.01, **** *p* ≤ 0.0001, compared to control.

In addition, Act d 1-treated HEK239 cells provided evidence for the upregulation of mRNA expression for the innate pro-allergenic cytokines IL-25 and TSLP (Figure 7).

**Figure 8:**
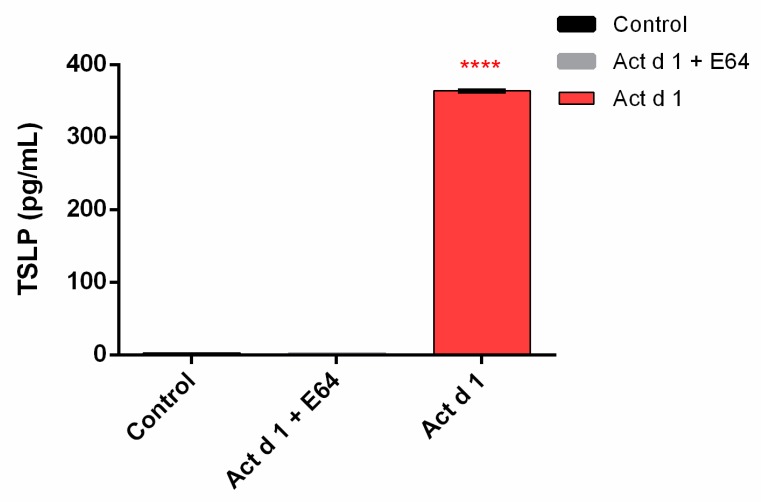
Act d 1 induces the release of TSLP from HEK293 cells. Results are expressed as mean ± SEM and analyzed using a one-way analysis of variance (ANOVA). **** *p* ≤ 0.0001 compared to control.

ELISA was employed to explore the effect of Act d 1 on HEK293 epithelial cells in terms of the production of pro-inflammatory cytokines. The 6-h treatment induced a significant release of TSLP (Figure 8).

## 4. Discussion

Disruption of the intestinal barrier has been associated with food allergy [23]. This is especially the case with food proteases, which are capable of inducing changes in the morphology of the epithelial barrier and of affecting intestinal permeability [19,24]. Act d 1 is an abundant allergen in kiwifruit that preserves its proteolytic activity in the intestine [19,25]. Experimental data indicates that Act d 1 could exert direct proteolytic cleavage of the tight junction proteins [19]. The molecular mechanism behind this effect has not been clarified.

In this study, increased intestinal permeability to BLG was observed in mice after oral administration of active Act d 1. These findings are in consistence with our previous results on increased permeability of the mouse intestine for 40 kDa FITC-dextran upon Act d 1 treatment [19] and with other studies showing an increase in epithelial permeability and disruption of the TJs [26]. Tight junctions provide cell-to-cell adhesion in enterocytes and play a key role in the regulation of the barrier permeability, which varies in different segments of the intestines [27,28]. Three types of transmembrane proteins are in common to all TJs: claudins, MARVEL domain proteins, and junctional adhesion molecules (JAMs). Among those transmembrane proteins, occludin (OCLN) and claudins (CLDNs) form a barrier at the apical-lateral membrane of the cell while peripheral membrane proteins, like zonula occludens (ZO) proteins, serve as a link between the transmembrane TJ proteins and the cytoskeleton [29]. Claudins form selective pores for the paracellular transport and, hence, are the most important proteins involved in the permeability of the intestinal barrier [30,31]. By immunofluorescence staining, it was shown that CLDN1, −3, and −4 are dominantly expressed in the colon while CLDN2 is predominantly expressed in the small intestine of mice [23]. In addition, CLDN2 is the main pore-forming protein and its expression is strongly upregulated in the duodenal biopsies of patients with food allergy [27,32,33]. Our results clearly demonstrate that mRNA expression of CLDN2 and E-cadherin in the proximal small intestine of mice is significantly upregulated upon Act d 1 administration. This could be a compensatory effect, which is an indication of the altered integrity of the intestinal epithelial cell layer [34].

Expression of CLDN1 was previously found to be decreased in the distal small intestine of patients with food allergy [27,35]. Our findings are in line with this observation and show that expression of CLDN1 was downregulated both in the distal small intestine and in the colon upon Act d 1 treatment. mRNA expressions of CLDN4, OCLN, and ZO1 were significantly decreased in the proximal part of the small intestine, while mRNA expressions of CLDN1 and CLDN4 were downregulated in the distal small intestine. In the colon, the opposite effect was observed, with increased expressions of CLDN3, CLDN4, OCLD, and ZO1 and decreased CLDN2 expression. Overall, our results from the in vivo study indicate the claudin family of TJs as the most affected by Act d 1 treatment in the proximal small intestine and in the colon.

The epithelium is crucial for the initiation and orchestration of immune responses leading to sensitization to allergens. This includes secretion of pro-allergenic cytokines such as IL-33, IL-25, and TSLP [14,36]. In this study, we found that depending on the location in the intestine, IL-33, IL-25, and TSLP mRNA were increased after administration of Act d 1.

The role of food proteases in the development of Th2 immune response correlates with increased IL-25 and TSLP gene expression [37]. By employing an in vitro model HEK293 cell system, we showed that Act d 1 can affect the integrity of HEK293 epithelial cell monolayer and decrease TEER. Act d 1 upregulates the mRNA expression of IL-25 and TSLP but also activates the NF-ĸB signaling pathway. In HEK293 cells, proteolytically active Act d 1 induced secretion of TSLP, an important gut epithelial cytokine, which can activate Th2 cells.

## 5. Conclusions

In summary, we provide a model where exposure of the mouse intestine to a food proteolytic enzyme compromised the integrity of the epithelial barrier and mediated upregulation of pro-Th2 cytokines, possibly via NF-ĸB. Increased epithelial permeability in the gastrointestinal tract is a risk factor for various antigens from the lumen to reach the submucosa and to induce inflammation. These findings provide evidence that food proteins with proteolytic activity can trigger innate immunity. Improvement of the mechanistic understanding of food allergy should pave a way for the better management of food allergies. The importance of these findings is a better insight into molecular events that contribute to the breakdown of clinical and immunological tolerance to kiwifruit, allowing the design of future therapeutic approaches in the management of food allergy.

## Figures and Tables

**Table 1:** Murine primer sequences used in *q*RT-PCR.

Target Gene (Reference)	Murine Primer Sequence (5′–3′)	Tm (°C)
CLDN1 (NM_016674.4)	F: TCTACGAGGGACTGTGGATG	57
	R: TCAGATTCAGCTAGGAGTCG	
CLDN2 (NM_016675.4)	F: GGCTGTTAGGCTCATCCAT	55
	R: TGGCACCAACATAGGAACTC	
CLDN3 (NM_009902.4)	F: AAGCCGAATGGACAAAGAA	58.7
	R: CTGGCAAGTAGCTGCAGTG	
CLDN4 (NM_009903.2)	F: CGCTACTCTTGCCATTACG	55
	R: CTGGCAAGTAGCTGCAGTG	
OCLN (NM_008756.2)	F: ATGTCCGGCCGATGCTCTC	61.2
	R: TTTGGCTGCTCTTGGGTCTGTAT	
ZO1 (NM_009386.2)	F: CGAGGCATCATCCCAAATAAGAAC	58.7
	R: TCCAGAAGTCTGCCCGATCAC	
E-cadherin (NM_009864.2)	F: ACTGTGAAGGGACGGTCAAC	64.3
	R: GGAGCAGCAGGATCAGAATC	
IL-33 (NM_001164724.1)	F: GGTGTGGATGGGAAGAAGCTG	61
	R: GAGGACTTTTTGTGAAGGACG	
TSLP (NM_021367.2)	F: CGGATGGGGCTAACTTACA	61
	R: TCCTCGATTTGCTCGAACTT	
IL-25 (NM_080729.3)	F: CAGCAAAGAGCAAGAACC	61
	R: CCCTGTCCAACTCATAGC	
ACTB (NM_007393.3)	F: ATGCTCCCCGGGCTGTAT	61
	R: CATAGGAGTCCTTCTGACCCATTC	

**Table 2:** Human primer sequences used in PCR.

Target Gene (Reference)	Human Primer Sequence (5′–3′)	Tm (°C)
TSLP (NM_009903.2)	F: CGCTACTCTTGCCATTACG	55
	R: ACTCAGCACACCATGACTTG	
IL-25 (NM_022789.3)	F: CCAGGTGGTTGCATTCTTGG	49
	R: TGGCTGTAGGTGTGGGTTCC	
IL-33 (NM_033439.3)	F: CACCCCTCAAATGAATCAGG	51
	R: GGAGCTCCACAGAGTGTTCC	
GAPDH (NM_009386.2)	F: CGAGGCATCATCCCAAATAAGAAC	58.7
	R: TCCAGAAGTCTGCCCGATCAC	
